# Heterogeneity in pragmatic randomised trials: sources and management

**DOI:** 10.1186/s12916-022-02569-w

**Published:** 2022-10-28

**Authors:** Bruno Giraudeau, Agnès Caille, Sandra M. Eldridge, Charles Weijer, Merrick Zwarenstein, Monica Taljaard

**Affiliations:** 1 Université de Tours, Université de Nantes, INSERM, SPHERE U1246, 2 Bd Tonnellé, 37044 Tours cedex 9, France; 2 INSERM CIC1415, CHRU de Tours, Tours, France; 3 Centre for Primary Care and Public Health, Queen Mary University of London, 58 Turner Street, London, E1 2AB UK; 4 Departments of Medicine and Philosophy, Western University, Stevenson Hall 4130, 1151 Richmond Street, London, ON N6A 5B7 Canada; 5 Centre for Studies in Family Medicine, Department of Family Medicine Schulich School of Medicine & Dentistry Western University, 1151 Richmond Street, London, ON N6A 3K7 Canada; 6 Clinical Epidemiology Program, Ottawa Hospital Research Institute, The Ottawa Hospital, Civic Campus, 1053 Carling Avenue, Ottawa, Ontario K1Y 4E9 Canada; 7 School of Epidemiology and Public Health, University of Ottawa, Ottawa, Canada

**Keywords:** Pragmatic randomised trials, Heterogeneity, Cluster randomised trials

## Abstract

**Background:**

Pragmatic trials aim to generate evidence to directly inform patient, caregiver and health-system manager policies and decisions. Heterogeneity in patient characteristics contributes to heterogeneity in their response to the intervention. However, there are many other sources of heterogeneity in outcomes. Based on the expertise and judgements of the authors, we identify different sources of clinical and methodological heterogeneity, which translate into heterogeneity in patient responses—some we consider as desirable and some as undesirable. For each of them, we discuss and, using real-world trial examples, illustrate how heterogeneity should be managed over the whole course of the trial.

**Main text:**

Heterogeneity in centres and patients should be welcomed rather than limited. Interventions can be flexible or tailored and control interventions are expected to reflect usual care, avoiding use of a placebo. Co-interventions should be allowed; adherence should not be enforced. All these elements introduce heterogeneity in interventions (experimental or control), which has to be welcomed because it mimics reality. Outcomes should be objective and possibly routinely collected; standardised assessment, blinding and adjudication should be avoided as much as possible because this is not how assessment would be done outside a trial setting. The statistical analysis strategy must be guided by the objective to inform decision-making, thus favouring the intention-to-treat principle. Pragmatic trials should consider including process analyses to inform an understanding of the trial results. Needed data to conduct these analyses should be collected unobtrusively. Finally, ethical principles must be respected, even though this may seem to conflict with goals of pragmatism; consent procedures could be incorporated in the flow of care.

## Introduction

Heterogeneity refers to the general concept of variability. In clinical studies, we classically consider three different types of heterogeneity [1]: clinical heterogeneity or “variability in participants, interventions and outcomes”, methodological heterogeneity or “variability in study design and risk of bias” and statistical heterogeneity or “variability in the intervention effects being evaluated in different studies”. Here, we focus on clinical and methodological heterogeneity, limiting ourselves to within-trial heterogeneity.

In 1967, Daniel Schwartz and Joseph Lellouch developed the concepts of explanatory and pragmatic attitudes in randomised clinical trials [2]. The explanatory approach “aim[s] at understanding. It seeks to discover whether a difference exists between two treatments which are specified by strict and usually simple definitions.” In contrast, the pragmatic approach “aim[s] at decision. It seeks to answer the question—which of the two treatments should we prefer?” Pragmatic trials aim to generate evidence to inform decisions made by patients or participants, physicians or other providers and health-system managers or other policy-makers [3]. Thus, a pragmatic trial must reproduce as much as possible the circumstances—including heterogeneity—under which the assessed intervention would be used in usual care. Pragmatic trials may be individually randomised or cluster randomised [4]. A cluster randomised trial is a trial in which intact social units rather than individual participants are randomised [5]. The units can be clinical (e.g. practices, wards, caregivers) or not (e.g. schools, geographical areas, families).

Because a pragmatic trial is expected to emulate usual health care delivery in the target setting, it should mimic the heterogeneity in patient outcomes expected outside the trial context. As a consequence, when planning, conducting and analysing a trial, some forms of heterogeneity should be welcomed (because they contribute to the fact that the trial mimics the future reality), but others are undesirable (because they are induced by the trial context and are not expected to be encountered in the future reality). In this paper, we aimed to identify these desirable and undesirable sources of heterogeneity in pragmatic trials based on our opinion. For each of them, we also discuss and illustrate with examples how they should be handled in trial planning, conduct and analysis to help people conduct their trials in a way to support pragmatic aims. Our analysis is based on the expertise and judgements of the authors consisting of four senior biostatisticians, a bioethicist, and a pragmatic trialist, all with a long experience in randomised trials.

According to the Patient, Intervention, Comparison, Outcome and Setting (PICOS) framework, [6] the manuscript is structured in three sections: (1) patients and settings of included centres (P and S domains of the PICOS), (2) intervention and control (C and O domains of the PICOS), and (3) outcome (O domain of the PICOS)), to which we added a fourth section related to regulatory and ethical issues, which may also affect heterogeneity. Table 1 summarises sources of heterogeneity in pragmatic trials and Table 2 our recommendations for management.

**Table 1:** Sources of heterogeneity in pragmatic trials as compared to explanatory trials

Source of heterogeneity	Explanatory trial	Pragmatic trial
Patients and setting of included centres	May try to limit enrolment to academic centres and centres with a more homogeneous patient case mix. Usually have a large number of selection criteria, some of which may be designed to include a homogeneous group of patients more likely to respond to treatment, adhere to treatment and complete follow-up.	Deliberately include a variety of centres, possibly with a heterogeneous patient case mix. Usually have fewer selection criteria so as to deliberately include a diversity of patients who will qualify for the treatment in clinical practice.
Intervention and control	Treatments are highly protocolised; enforce strict adherence to the protocol in each arm; co-interventions are not allowed or are limited and specified in detail; blind patients and providers to eliminate performance bias and subjectivity in assessment of outcomes.	Permit some tailoring while keeping core interventions common across all sites and for all participants; do not enforce adherence to protocol; permit co-interventions that would be used in target sites and settings after the intervention is shown to be effective; avoid using placebo; avoid blinding patients or providers.
Outcome	Standardised outcome assessment; use central adjudication; blind outcome assessors to eliminate subjectivity in assessment of outcomes.	Favour objective outcomes, relevant for both patients and physicians; standardisation, adjudication and blinding of outcome assessors should be discouraged except if there is a risk of biasing the trial result.
Regulatory and ethical issues	Requirement for written, informed consent from all participants; vulnerable participants (e.g. patients with co-morbidities or lacking decision-making capacity) commonly excluded.	In some cases, “clinical-style” or integrated consent may be permitted. Vulnerable participants generally included.

**Table 2:** Authors’ recommendations for managing sources of heterogeneity in pragmatic trial design, conduct and analysis

Trial design	Trial conduct	Trial analysis
**Sources: Centres and patients**		
• Ensure that centres where results are intended to be applied are clearly defined. • Attempt to recruit centres typical of target population (rather than convenience sample). • Increase number of centres (possibly at cost of decreasing sample size per centre). • Ensure that patient populations to whom results are intended to apply are clearly defined and reflected in selection criteria, which should not be too restrictive. • Stratify by centre in randomisation, whenever possible. • Consider stratifying randomisation by other important prognostic factors. • Sample size may need to be increased to reflect pragmatic features, including potentially attenuated intervention effect and larger variance estimate. • In cluster randomised trials, ensure that intracluster correlation and cluster size variation are taken into account.	• Inclusion criteria should be applied consistently across all centres and investigators.	• Include stratification factors in analysis. • Limit number of planned subgroup analyses to those relevant to usual clinical decision-making.
**Sources: Intervention and control**		
• Implement the trial such that, aside from the intervention, the trial has the lowest possible impact on patients and caregivers so as not to distort usual care conditions. • Consider likely extent of non-adherence, contamination, and co-interventions when specifying effect size for sample size calculation.	• Allow tailoring but maintain core intervention features that define the intervention being assessed. • Avoid interventions to monitor and promote compliance that may change patient behaviour unless they can be incorporated into future scale-up of intervention. • Assessment of compliance (by centres, providers, and patients) should be incorporated into intervention or assessed as a secondary outcome but measured in an unobtrusive way that does not interfere with compliance that would be expected outside the trial.	• Conduct analysis of superiority trials by intention-to-treat. • Ancillary process analyses can shed light on understanding mechanisms of observed treatment effects as long as data to facilitate such analyses are collected unobtrusively.
**Sources: Outcomes**		
• Ideally, outcomes should be selected to be relevant to patients or trial stakeholders and assessed in usual care. • Avoid blinding outcome assessors except if there is a high risk of detection bias; select an outcome as objective as possible. • If needed, standardise how the outcome is assessed and adjudicate it.	• Sensitise data monitoring committees to the pragmatic nature of the trial to prevent undue intrusion through excessive data collection.	
**Sources: Regulatory and ethical issues**		
• Plans for recruitment and informed consent must adhere to internationally accepted ethical principles even though this may affect pragmatism and bias. • Inclusion of vulnerable participants is appropriate provided plans are in place to identify and protect those who cannot provide informed consent and who may be at risk because of co-morbidities. • “Clinical-style” or integrated consent may be appropriate for recruitment in clinical settings when interventions involve usual care. • Waiver of consent may be requested from a research ethics committee when a cluster-level intervention poses only minimal risk.		

## Patients and setting of included centres

### Trial planning: select typical centres

Centres involved in a pragmatic trial should be drawn from a similar range of patient care settings as those in the target population for which the designers intend the findings of the trial will apply [7]. If study centres are limited and highly selected, heterogeneity will be reduced and may no longer fit the target population. For instance, centres should not exclusively be university hospitals when the disease of interest is common, and patients are cared for in both community and university hospitals (e.g. NUTRIREA-2 trial [8], Table 3).

**Table 3:** NUTRIREA-2: enteral versus parenteral early nutrition in ventilated adults with shock

**Patients**: Adults receiving invasive mechanical ventilation and vasoactive drugs for shock **Centres**: French intensive care units **Intervention**: Enteral nutrition **Control**: Parenteral nutrition **Outcome**: Day 28 all-cause mortality **Design**: Two parallel-groups, individually randomised trial	
**Centre selection**	Both community and university care hospitals were recruited: “44 French ICUs, including 28 (64%) in university hospitals”
**Randomisation**	“Randomisation was stratified by centre using permutation blocks of variable sizes”

An option is to maximise the number and range of included centres, perhaps reducing the number of patients per centre. In trials conducted across a health system, it may even be possible to recruit centres in random sequence until the required sample size is reached, thereby vouchsafing representativeness of the available sample and thus applicability to the target population (e.g. IRIS trial [9], Table 4).

**Table 4:** IRIS: training program to increase identification of female victims of domestic violence

**Participants**: Women aged over age 16 **Centres:** General practices **Intervention**: Practice-based training sessions and pop-up template in electronic medical record **Control**: Usual care **Outcome**: Number of referrals **Design**: Two parallel-group, cluster randomised trial, clusters being general practices	
**Centre selection**	“To ensure inclusion of practices with a range of characteristics, we stratified them by four characteristics (proportion of whole time equivalent female doctors, general practice postgraduate training status, number of patients registered with the practice, and percentage of the practice population on low incomes defined by the low income scheme index), then ordered them randomly within strata and invited them to participate in the trial sequentially within each strata by email or letter.”
**Variability in cluster (centre) size**	“With 24 intervention practices and 24 control practices, with the assumption of an identification rate of 1% in control practices (a conservative estimate based on our survey of 12 east London practices) and an intracluster correlation coefficient of 0·03, we would be able to detect a difference of 5·2% in the identification rate with a power of 80% at a significance level of 0·05. This calculation assumed an average of 1600 women in the relevant age group in every practice, and took account of variation in cluster size.”
**Randomisation**	“To ensure inclusion of practices with a range of characteristics, we stratified them by four characteristics (…).” “Within every primary care trust area we randomised practices with a computer minimisation programme, with a random component (Minim Version 1.3), maintaining allocation concealment. JR ran the minimisation programme for every practice after they were recruited and then informed the research associates of the allocation. The minimisation variables were the same as the stratification variables.”
**Statistical analysis**	“Analysis was done for all practices for which we obtained baseline data, adjusted for minimisation factors (…).”

In a cluster randomised trial, heterogeneity in selected centres has two further consequences. First, more variability in outcome between centres increases the intraclass correlation coefficient, and as a result, a larger sample size is required. Second, variability in cluster (centre) size also increases the required total sample size [10].

Finally, although differences in patient characteristics between centres may reflect a different patient case-mix between centres [11], which is a welcome source of heterogeneity, such differences may also be due to the differential application of eligibility criteria, which is an undesirable source of heterogeneity [12]. Indeed, in a cluster randomised trial, such a phenomenon would be a source of bias because of differences in characteristics of included participants between the groups being compared; in an individually randomised trial, this situation may induce a centre effect, which would not be due to the intervention but rather to differences in following the trial procedures.

### Trial planning: relax patient selection criteria

A pragmatic trial aims to recruit patients from an available population who are as similar as possible to the target population. This target population corresponds to the population that would receive the study intervention once it has been shown to be effective and scaled up in the usual healthcare setting. Eligibility criteria should not exclude patients who are less likely to respond to the treatment or those not likely to complete the follow-up. Success in representing the target population in the patients recruited for the trial contributes to the applicability of the trial’s results [13] to the target population. Inclusion and exclusion criteria are often more restrictive in trials of drug interventions than those assessing devices, surgery or other complex interventions; they are also more restrictive in industry-sponsored versus public agency-funded trials [14]. As an example, the TiME trial [15] had very few selection criteria for patients, thus promising very good applicability, besides the fact that it limited the risk of identification and recruitment bias (Table 5).

**Table 5:** TiME: increased haemodialysis duration session

**Patients**: Adults with thrice-weekly in-centre haemodialysis (see below) **Centres**: Dialysis facilities **Intervention**: Haemodialysis session duration of ≥ 4.25 h **Control**: Usual care **Outcome**: Mortality **Design**: Two parallel-group cluster randomised trial, clusters being haemodialysis facilities	
**Patient selection criteria**	“Inclusion criteria for patients were (1) age ≥18 years, (2) treatment with thrice-weekly in-center hemodialysis, and (3) initiation of dialysis within the previous 120 days. Exclusion criteria for patients were (1) use of a health care proxy to provide consent for dialysis treatment and (2) unwillingness to have clinical data included in the trial dataset.”
**Randomisation**	“Dialysis facilities were randomised 1:1 to the intervention or the usual care group, using a permuted block randomisation procedure with stratification by dialysis provider organization, and by factors known to be associated with mortality: racial composition (≤50% or >50% black patients) and use of central venous catheters for hemodialysis vascular access (≤20% or >20% of patients).”
**Compliance**	“Participant follow-up ended on January 31, 2017 on the basis of the recommendation by the DSMB to terminate the trial because of a lower than anticipated difference in session duration between the intervention and usual care groups (…).” “For the primary analysis population, the estimated mean prescribed session duration was 219 (95% confidence interval [95% CI], 217 to 222) minutes in the intervention group and 210 (95% CI, 209 to 213) minutes in the usual care group.” “Discussions with facility staff and medical directors during the course of the trial indicated that the major reasons for poor uptake of the intervention were unwillingness by patients to have longer dialysis treatments, perception by the treating nephrologists that longer dialysis was not needed because of adequate solute clearance, and perception by the treating nephrologists that longer session durations were not in the best interest of a patient because of older age and/or frailty.”

### Trial planning: account for pragmatic features in sample size calculation

Even though sample size formulae may be the same, the reasoning about sample size differs in pragmatic and explanatory trials. First, intervention effects are expected to be smaller in pragmatic than explanatory trials, in part because of the inclusion of patients with a wider range of characteristics, for example those with comorbidities, who are less adherent, and/or who have both less severe conditions, and thus benefit less, as well as those whose condition is more severe and possibly intractable. Other features that might promote homogeneity and thus apparently greater effect sizes in explanatory trials include selecting caregivers and centres based on volume and experience [16]. Second, sample size parameters need to be carefully and realistically specified. A priori specifying a standard deviation that is lower than the post hoc estimate is a common problem [17] and results in optimistic sample size estimates and risks of insufficient statistical power. Therefore, attention should be paid to whether standard deviation estimates are derived from previously conducted explanatory trials—and therefore likely to be too low—or from administrative routinely collected data, for instance, which should adequately capture real-world heterogeneity.

### Trial planning: stratify randomisation

A centre effect is to be expected in a pragmatic trial because of centre and participant heterogeneity, as previously discussed. The intervention delivery may also be tailored to the centre context, and such heterogeneity, which, in our opinion, should be welcomed because interventions are applied with heterogeneity in real practice, also contributes to a centre effect. Accordingly, to prevent imbalance between arms and improve power, individually randomised multicentre pragmatic trials should stratify randomisation on centre [18] (e.g. NUTRIREA-2 trial, Table 3). Prognostic factors may also be considered as stratification variables (e.g. ALIC^4^E trial [19], Table 6), notably when the sample size is small, thus limiting the risk of baseline imbalances [20]. Similarly, for cluster randomised trials, restricted randomisation such as, for instance, stratified randomisation or randomisation by minimisation, is advisable to limit chance imbalances (e.g. IRIS and TiME trials, Tables 4 and 5) [5].

**Table 6:** ALIC^4^E: oseltamivir in patients with influenza-like illness

**Patients**: Both adults and children with symptoms of influenza-like illness **Centres**: Medical practices that were part of primary care research networks **Intervention**: Oseltamivir plus usual primary care **Control**: Usual primary care **Outcome**: Time to recovery **Design**: Two parallel-group individually randomised trial	
**Randomisation**	“Stratified block randomisation was implemented, with random blocks of two, four, and six participants and stratification by age (< 12, 12–< 65, and ≥ 65 years), overall severity of influenza like illness (rated by the responsible clinician as mild, moderate, or severe), any relevant comorbidity (yes or no for heart disease, diabetes, chronic respiratory condition, hepatic, haematological, neurological, or neurodevelopmental condition, stroke or transient ischaemic attack, or overnight hospital stay in previous year), and previous duration of symptoms since onset (≤48 h or >48–72 h, based on recommendations that oseltamivir should be started within 48 h of symptom onset).”
**Blinding**	“This was an open-label study, so no placebo was used and drugs were not masked.” “Some might consider the absence of a placebo control as a limitation. We deliberately chose to do an open-label trial in the context of everyday practice, because effect sizes identified by placebo-controlled, efficacy studies with tight inclusion criteria might not be reproduced in routine care. We also wished to estimate time to patient reported recovery from the addition of an antiviral agent to usual care rather than benefit from oseltamivir treatment compared with placebo. This pragmatic, open trial design makes our findings likely to reflect real world effects in primary care, because knowledge of what medication one is taking could affect subsequent help seeking and health behaviour and use of symptomatic medications. However, the design did not allow us to be sure of mechanisms or how much of the observed effect can be attributed to specific oseltamivir or other possible effects, and the relative contribution of such possible effects which might differ for the various subgroups.”

### Trial analysis: adjust on stratifying variables, notably centres (e.g. IRIS trial, Table 4)

Although not specific to pragmatic trials, unadjusted analyses of trials using stratified randomisation raise two issues. First, there is inconsistency if factors used to stratify randomisation are not taken into account when analysing the results. Second, ignoring stratification factors in the analysis leads to over-estimated standard errors, wider confidence intervals, inflated *p*-values and diminished power [21]. Although this is true for any randomised trial, it is a particular concern in pragmatic trials in which between-centre heterogeneity is expected to be higher, as discussed above. Accounting for centre effects is therefore advisable ant it has been shown that random-effects models offer better properties than fixed-effects models [21].

### Trial analysis: limit subgroup analyses to those that inform decision-making

Subgroup analyses aim to identify interactions between treatment and pre-specified patient or centre characteristics [22]. Because pragmatic trials aim at informing decision-making rather than promoting an understanding of the mechanism of action, subgroup analyses should only be done if the same subgroups are meaningfully part of usual clinical care or policy decision-making, which requires that the distinction between these subgroups is readily accessible to clinicians (e.g. age, blood pressure), (e.g. APTS trial [23], Table 7) or policy-makers (e.g. subgroups defined by equality, diversity, and inclusion groups.

**Table 7:** APTS: Delayed cord clamping

**Patients**: Fetuses from women expected to deliver before 30 weeks of gestation **Centres**: 25 centres in seven countries **Intervention**: Delayed cord clamping **Control**: Immediate cord clamping **Outcome**: Composite outcome of death or major morbidity **Design**: Two parallel-group individually randomised trial	
**Sample size calculation: non-adherence**	“The original sample was 1600 infants, yielding 90% power (two-sided P = 0.05) to detect an absolute difference in the incidence of the primary outcome of 8 percentage points between the two groups (30% in the immediate-clamping group vs. 22% in the delayed-clamping group; relative difference, 27%), with the assumption of 10% nonadherence. If the rate of nonadherence to the Intervention and loss to follow-up reached 20%, there was more than 80% power to detect this difference.”
**Subgroup analysis**	“Tests for interaction were used to detect heterogeneity for the primary outcome in three prespecified subgroups: gestational age (<27 weeks vs. ≥27 weeks), sex, and method of delivery (cesarean section vs. vaginal delivery).”

## Intervention and control groups

### Trial planning: permit some tailoring of the intervention

Although heterogeneity in the delivery of interventions is an undesirable feature of an explanatory trial (in which interventions must be standardised), in pragmatic trials, as in future usual care in the target settings, interventions may well be tailored to individual patient needs or the local context in which care is provided [24], especially for complex interventions [25] (e.g. OPERA Trial [26], Table 8).

**Table 8:** OPERA: physical activity to prevent depression in residential homes

**Patients**: Care home residents aged ≥ 65 years **Centres:** Care homes from Coventry and Warwickshire and northeast London **Intervention**: Depression awareness programme delivered by physiotherapists, plus physical activity programme (see below) **Control**: Depression awareness programme for care home staff **Outcome**: Prevalence of depression **Design**: Two parallel-group cluster randomised trial, clusters being residential care homes	
**Intervention**	“On the basis of the assessment the physiotherapist determined a plan of action for the intervention programme elements. The first was a bespoke physical activity programme tailored to each resident and aimed at increasing the level of habitual physical activity, developed in co-operation with the physical activity champion/senior carers. This included the provision of mobility aids, advice on footwear, and manual handling tips to enable mobility. The second was to determine the appropriate level of exercise activities for the group exercise programme.”
**Process analysis**	“Alongside the main study we carried out a process evaluation and long-term follow-up using both qualitative and quantitative methodologies to explore the process of implementing the study in a care home setting to develop a set of transferable principles regarding both the OPERA depression awareness training and the OPERA ‘whole-home’ exercise intervention to inform its implementation on a wider scale. We did independent observations of the process of obtaining consent from participants. We did focus groups and interviews with key informants about the process of consent in care home studies.”

Hawe et al. refer to standardisation by function as compared with standardisation by form (e.g. rather than using a common information kit, how information is provided may differ among centres while the function of the information remains constant across centres), acknowledging that mechanisms that are assessed (i.e. the very components of the intervention) can take different forms from one context to another [25]. Nevertheless, the core components of an intervention need to be specified [27]; otherwise, the interpretation of the results may be complex because one would not know what intervention is being evaluated.

Tailored interventions may contribute to a centre effect [18] or even a provider effect [28], but depending on the research question and trial intention, flexibility in interventions is relatively unproblematic as long as in the trial interventions are delivered by providers in a similar range of ways and in settings that match the target clinical settings. Doing so will introduce desirable heterogeneity in participant outcomes because it mimics reality in that interventions are rarely perfectly standardised in usual care.

Monitoring the extent of tailoring as well as co-interventions raises a further dilemma. On one hand, we want to better understand what actually happened, and this knowledge may help to scale up the intervention after the study has demonstrated benefit. This is the very aim of a process analysis, which is both desirable and recommended [29] (e.g. OPERA Trial, Table 8). On the other hand, any intrusive data collection is undesirable, because it may distort usual clinical practice and patient response. Indeed, patient and health provider behaviour should not be altered outside of the provision of the intervention, to limit as much as possible a Hawthorne effect [30]. Ideally, process measures and outcome assessments should be as unobtrusive as possible, perhaps obtained using administrative or electronic medical record data whose collection is part of the usual care.

### Trial planning: ensure that the control intervention reflects usual care

Control interventions are typically non-protocolised usual care or, in comparative effectiveness research, another already widely used active treatment. The use of a usual-care control has several consequences. First, the control can be “no treatment,” but it should rarely be a placebo [31] because placebos are not used in usual clinical care outside of trial contexts. This unnatural comparison group may alter the results of the trial in unknowable ways. Moreover, a placebo control could contribute to an unnatural and undesirable homogeneity among patients allocated to the control group, by reducing recourse to self-prescription with medicines or other treatment modalities (e.g. ALIC^4^E trial, Table 6). It may also affect outcome assessment, which raises other issues, notably related to the risk of detection bias (cf. Outcome section). We acknowledge that not using a placebo may be a challenging issue for a regulatory agency and therefore, if relevant, encourage trialists to have preliminary discussions with these agencies to justify the need for avoiding placebos. Second, there may be different approaches to usual care in different centres of the target setting. This situation may be accommodated by more than one control group or a single control group that permits unrestricted implementation of a variety of different treatments used in routine care and thus averages out all the kinds of usual care provided [32]. Third, a usual care control means that we expect patients and providers to behave as they would outside a trial context. However, for both patients and providers, behaviours can be altered by trial enrolment, known as the Hawthorne effect [30]. Changes in patient and provider behaviours may affect patient outcome heterogeneity, probably by reducing it. This raises an unsolvable conundrum: except in rare situations, which must be approved by an ethics committee, both patients and providers must be informed that they are involved in a randomised controlled trial. This information procedure is a mainstay of ethical clinical research but may alter behaviours as compared with usual, unobserved, non-trial care. This situation is a strong argument for incorporating consent procedures in the flow of care [33], minimising the obtrusiveness of intervention and data collection in order to minimise participant awareness of the trial and thus minimise the Hawthorne effect.

### Trial planning: consider the impact of compliance on sample size

Lack of compliance is common outside a trial context. Sample size calculation should take into account usual-care levels of compliance [34] (e.g. APTS trial, Table 7). Moreover, in pragmatic trials comparing usual-care interventions without blinding, patients from one group may sometimes be easily able to access another study group intervention, which may result in contamination. If this contamination is symmetrical between arms, then it increases variability and decreases the effect size estimate. If this contamination is not symmetrical between arms, which is the most plausible situation, it creates a bias, which can attenuate or exaggerate the effect size estimate. In both situations, the issue cannot be dealt with merely by increasing the sample size. Cluster randomisation may limit contamination, but it may also induce bias arising from the identification or recruitment of individual participants if this processes happen after randomisation [35]. This could be a worse problem than group contamination in the individually randomised version of that trial [36].

### Study conduct: do not enforce compliance

In explanatory clinical trials, compliance with intervention and control protocols by both providers and patients is enhanced by trial monitoring often followed by direct contact between a research assistant and the non-compliant patient or provider [37]. However, in pragmatic trials, efforts to promote compliance are undesirable unless such efforts are viewed as part of the intervention itself and would be scaled up in usual practice. The guiding principle is that outside of the study intervention—which should be provided similar to how it would be provided in future usual care should it be shown to be effective in this trial—other behaviours of providers and patients should be unaltered. Trial monitoring is deeply ingrained in the minds of both researchers and study sponsors and setting it aside when performing a pragmatic trial requires a paradigm change. Thus, in pragmatic trials, compliance should not be enhanced but rather considered an outcome and assessed unobtrusively [4]. In the TiME trial (Table 5), although the stated goal of pragmatism had been impaired owing to efforts made to enhance adherence and assess compliance, compliance turned out to be of major interest. Indeed, intervention fidelity was so poor that any difference between groups in haemodialysis session duration (the intervention assessed) vanished over time, which led authors to discontinue the trial.

### Study conduct: allow co-interventions

Co-interventions, defined as additional treatments that are not part of the assessed intervention, are another source of heterogeneity. In an explanatory trial, possible co-interventions are listed in the study protocol; some of these may be allowed, but others are prohibited. In a pragmatic trial, co-interventions are not generally considered protocol violations: they are left to the discretion of patients and providers in the trial because this flexibility would apply to usual care in the target setting, once the intervention is in widespread use, and where similar co-interventions will be in use. Measuring them is of interest, but it remains a secondary objective aimed at understanding, and as much as possible, it should be done in an unobtrusive way.

### Trial analysis: apply the intent-to-treat principle

Statistical analysis of a superiority trial is expected to be according to intent-to-treat, and this holds true for pragmatic trials [7, 38]. Indeed, per-protocol, completers, on-treatment or complier average causal effect (CACE) analyses aim at understanding what could be observed with optimal compliance and are more suited to explanatory trials [39]. Some argue that per-protocol analyses are of interest if the intervention is expected to be scaled up in settings where adherence to treatment is expected to be better than in the conducted trial [40]. However, this situation casts doubts on the representativeness of the selected settings. One may also argue that per-protocol or CACE analyses are of interest from a patient perspective because they may help patients decide between treatments, though the necessity for perfect compliance to achieve the effects in such analyses needs to be acknowledged. Thus, such analyses should remain secondary analyses.

Missing data is an important issue in intent-to-treat analysis. Missing data may be more prevalent in a pragmatic than explanatory trial in which monitoring is more stringent, except if data are obtained from well-completed medical or administrative registries [41]. Therefore, statistical methods to handle missing data, such as multiple imputation or covariate adjustment, should be used [42] (e.g. ACUDep trial [43], Table 9).

**Table 9:** ACUDep: acupuncture and counselling for depression

**Patients**: Adults with depression **Centres**: General medical practices **Interventions**: Acupuncture and counselling **Control**: Usual care **Outcome**: Depression prevalence assessed with the Patient Health Questionnaire 9 at 3 months **Design**: Three parallel-group individually randomised trial	
**Statistical analysis**	“Multiple imputation by chained regression was used for missing data using treatment group, baseline measures (PHQ-9, BDI-II, SF-36, EQ-5D Anxiety/Depression), and demographics (age and gender). The primary analysis was based on the imputed rather than raw data in order to take account of the profile of non-responders.”

### Trial analysis: make sure ancillary studies will not interfere with not imposing specific constraints on patients or physicians

As an ancillary objective of a pragmatic trial, one may seek to better understand the assessed intervention. Thus, at the end of the study, a process analysis “[that] explore[s] the way in which the intervention under study is implemented” [29] may bring a complementary view taking into account contextual issues [44] (e.g. OPERA trial, Table 8). In the same way, per-protocol [40] or CACE analyses may help explain whether lack of treatment effect is due to lack of compliance, whereas subgroup analyses may help identify subgroups of patients who benefit most from the treatment. In a pragmatic trial, all these analyses are generally secondary ones, which means that no specific effort should be made to collect additional data for them if that extra data collection jeopardises the primary purpose of the study, perhaps by distorting the clinical setting and adding extra investigations or disruptive data collection. However, pragmatic trials aim at answering the questions that decision-makers need answered, so one cannot exclude the possibility that subgroup analyses may be part of the primary objective, for example, to investigate aims relevant to health equity.

## Outcome

### Trial planning: select a routinely collected outcome regarded as important by clinicians and patients

In pragmatic trials, the primary outcome must be directly relevant to patients or the primary stakeholder because it needs to inform decision-making by patients, caregivers and policy-makers [2, 7]. The primary outcome of a pragmatic trial should ideally correspond to an outcome routinely assessed in usual care and is regarded as clinically important and therefore likely to influence providers’ decisions (e.g. TASTE Trial [45], Table 10).

**Table 10:** TASTE: thrombus aspiration in myocardial infarction

**Patients**: Adults with ST-segment elevation myocardial infarction **Centres**: 29 Swedish centres and 1 Icelandic coronary intervention centre **Intervention**: Thrombus aspiration followed by percutaneous coronary intervention **Control**: Percutaneous coronary intervention **Outcome**: All-cause mortality at 30 days (see below) **Design**: Two parallel-group individually randomised trial within cohort	
**Outcome**	“Data on mortality obtained from the national population registry”. “The concept of a trial design using a national registry as the basis for continuous enrolment and randomisation of all-comers is potentially limited by the lack of formal central adjudication of clinical events. Therefore, we have chosen all-cause mortality from the national complete mortality registry as the primary end point of the trial.”

### Trial planning: avoid standardisation, blinding and adjudication as much as possible

Outcome assessment raises a conundrum. Some suggest that standardisation (i.e. applying standardised measurement methods), blinding and adjudication should be avoided because they do not correspond to usual practice [7]. Standardisation aims at reducing heterogeneity in outcome assessment, whose consequence is mainly a loss in power. Heterogeneity in outcome assessment also increases the risk of misclassification, which, may be a source of bias [46, 47]. Standardisation may occur for outcomes derived from interviews [48] but also for clinical examinations [49] or even in electronic health records [50]. Blinding and adjudication also aim at reducing the risk of bias (e.g. RESTART Trial [51], Table 11).

**Table 11:** RESTART: antiplatelet therapy after stroke due to intracerebral haemorrhage

**Patients**: Adults surviving spontaneous intracerebral haemorrhage **Centres**: 122 hospitals in the UK **Intervention**: Antiplatelet therapy **Control**: Usual care **Outcome**: Recurrence of symptomatic intracerebral haemorrhage (see below) **Design**: Two parallel-group individually randomised trial	
**Outcome**	“Although we did not mask the assigned treatment to participants and physicians, the outcomes were objective and adjudicated masked to treatment allocation, which minimises bias.”

Problems arise mainly for non-objective outcomes. Subjective outcome assessment is indeed known to be potentially influenced by the beliefs, in relation to the treatments, of patients themselves, their caregivers or clinicians [52]. Moreover, in the absence of blinding, this influence may not be the same in the groups being compared. However, another view of this is that these subjective beliefs in relation to the effectiveness of interventions would be active in clinical practice, after the trial has shown one of the tested interventions as more effective and been implemented widely. In that case, the subjective beliefs in the intervention have been well captured in the trial and thus reflect the future usual-care situation accurately. In this quite common situation, eliminating the effect of subjective belief in the trial would eliminate necessary heterogeneity and result in an incorrect estimate of the effect size.

Actually, standardisation, blinding and adjudication do not have the same consequences. Although blinding as well as standardised data collection by researchers may indeed affect patient and care-provider behaviours, adjudication is less problematic because it can be performed after data collection, with blinding to the arm of the patient whose record is being assessed and therefore without bias. However, adjudication, as we most often know, is performed by outside and selected expert clinicians often using information or expertise not available to the clinician in usual care in some future setting. This might produce trial results that differ from results based on usual-care clinician assessments thus reducing the relevance of the trial for decision-making. Although this trial may not be biased (the finding is true for the patients and outcome measures of the trial), it is less applicable to the usual-care situation.

### Trial conduct: sensitise data-monitoring committee to the pragmatic nature of the trial

The data monitoring committee is expected to think differently when investigators have clearly articulated their intended goal of pragmatism [50]. The committee should pay more attention to protecting external applicability and avoiding co-interventions delivered by the research team (not the patient and care-provider co-interventions) that are not visible when reading the intervention description in the trial protocol. Depending on the unique circumstances of each trial and intervention being assessed, it may nevertheless keep its original function of monitoring for safety concerns.

Many pragmatic trials, especially of complex non-clinical interventions such as service delivery changes, may not collect data other than at the end of the trial, and so ongoing data monitoring is not relevant because the intervention is low risk. Hence, safety signals are considered unlikely and will not be formally monitored with trial data. This situation may suggest that instead of a data safety or monitoring committee, a more comprehensive trial management committee may be an appropriate supervisory structure, paying more attention to issues such as intervention implementation, patient and centre recruitment, although provision should be made for processes to deal with data confidentially should the need arise during the trial.

If ongoing safety data collection is planned for a pragmatic trial, unobtrusive data sources such as administrative and electronic medical record data may be preferred because they have no effect on the flow of care. However, collecting from these sources may also have substantial time-lags before reliable datasets are assembled and cleaned. Therefore, safety monitoring for acute intervention-related injury, requiring a quick turnaround for action, may have to depend on clinical suspicion. Because intensive safety monitoring may disrupt the usual flow of care, a highly pragmatic design may not be suitable for trials evaluating interventions whose side-effect profile is not yet clear.

## Ethical and regulatory issues

Any randomised trial, pragmatic or not, must be conducted in accordance with internationally accepted ethical principles and regulatory guidelines. The very aim of such principles is to protect the autonomy and welfare interests of the participants in clinical trials, and the need for protection is not debatable given horrendous and inhumane “research” such as the Nazi medical experiments and the Tuskegee syphilis study that litter the history of medical research [53]. Participant autonomy is protected by informed consent procedures. With this process, participants voluntarily agree to have a follow-up specific to the study, to potentially experience risk, and to have personal and potentially sensitive data used for the research. Additional protections may be required for people who are particularly vulnerable to potential risks (e.g. children, prisoners or pregnant women, even though there may be no known clinical reason for doing so [54]) and also people with diminished autonomy (e.g. children or adults lacking decision-making capacity).

Patients who refuse to participate in trials may differ from those who agree to enrol (e.g. the Beaver et al. trial [55], Table 12).

**Table 12:** Telephone follow-up after treatment for breast cancer

**Patients**: Women treated for breast cancer, with a low to moderate risk of recurrence **Centres**: 2 UK centres **Intervention**: Telephone follow-up **Control**: Traditional hospital follow-up **Outcome**: Psychological morbidity assessed notably by the mean state-trait score **Design**: Two parallel-group individually randomised trial	
**Difference between patients who agreed or refused to be included**	“Those who refused to take part differed from participants in study site, social class, and follow-up status. Patients at the specialist breast unit (71%) were more likely to want to participate than those at the district general hospital (61%, *χ*^2^ = 5.01, df = 1, *P* = 0.025), participants from higher social classes (professional occupations) were more likely to want to participate than those from lower social classes (*χ*^2^ = 15.77, df = 8, *P* = 0.046), and participants with three to 12 months between visits (67.7%, 70.6%) were more likely to participate than those on six monthly follow-up (58.1%, *χ*^2^ = 7.66, df = 2, *P* = 0.022). Time from diagnosis did not differ significantly for those who did or did not take part (*t* = − 0.26, *P* = 0.80); those who refused to take part were a median of 21 months from diagnosis.”

In the end, excluding potential participants because of lack of consent may lead to a situation in which the risk profile of included participants may differ from the risk profile of those who were excluded. This situation may reduce heterogeneity among participants, and therefore, the representativeness of the included participants and the applicability of the trial. As a consequence, the challenge in maintaining heterogeneous participants and providers and settings in pragmatic trials may require that trial designers collaborate with ethicists and research ethics committees to obtain a proper balance between protecting research participants while promoting the applicability of the trial findings, although ethical issues must prevail over scientific ones.

Heterogeneity may also be induced by differences in requirements from different research ethics committees, which is an undesirable type of heterogeneity [56] (e.g. PADIT Trial [57], Table 13). Indeed, in such a situation, a patient could be considered eligible and included in some centres but not in others. Such a situation has some similarities with one in which selection criteria would not be applied in the same way among centres, which, as previously discussed, is a source of undesirable heterogeneity. In some countries, centralised research ethics committees can provide a single review covering all participating centres, thus improving consistency and reducing unwanted between-centre heterogeneity.

**Table 13:** PADIT: prevention of arrhythmia device infection

**Patients**: Patients with an implanted medical device **Centres**: 28 Canadian centres **Intervention**: Incremental periprocedural antibiotics, i.e. pre-procedural cefazolin plus vancomycin, intraprocedural bacitracin pocket wash, and 2-day post-procedural oral cephalexin **Control**: Conventional periprocedural antibiotics, i.e. pre-procedural cefazolin infusion **Outcome**: 1-year hospitalisation for device infection **Design**: Cluster randomised cross-over trial	
**Ethical requirements**	“All centers’ ethics boards approved the trial with waiver of consent for treatment. Ten centers required patient consent for data collection, which was generally obtained during follow-up.”

### Trial planning: inclusion of vulnerable patients and informed consent

Although vulnerable patients, including those with co-morbidities, are commonly excluded in explanatory trials, a more inclusive approach may be adopted in pragmatic trials, provided adequate protections are in place. For patients with co-morbidities, protections may include flexibility in administration of the study intervention to meet individual patient needs (e.g. dose reduction) and additional clinically indicated follow-up visits. When patients have diminished capacity to provide consent, a surrogate decision-maker may be required. This may also be the case for emergency research such as trials conducted in intensive care units.

Written informed consent for trial participation is standard for explanatory trials. Pragmatic trials are commonly conducted in primary care settings and usually involve routine medical interventions. Although the ethical principle of respect for persons requires that the autonomy of participants be respected, a more clinical approach to consent in pragmatic trials may achieve the same goal with less intrusion (and thus less propensity to increase homogeneity). Kim et al. [33] describe one such clinical approach to consent called “integrated consent”, whereby informed consent to participation in a pragmatic trial is sought by the health provider in the clinic, during the usual course of care delivery. The health provider discloses key features of study participation verbally and records the patient’s consent or refusal in the electronic health record. In a cluster randomised trial, when the study intervention is a cluster-level intervention (thus, indivisible at the level of the individual) and poses only minimal risk to participants, research ethics committees may grant a waiver of consent when the science would be compromised by seeking consent [58].

## Conclusion

Heterogeneity is a prevalent feature of all trials and may be more marked in pragmatic trials, which are expected to closely emulate the target settings. Between-patient variability is probably the main source of heterogeneity. However, there are many other sources of heterogeneity. Some are undesirable and therefore should be limited, but the pragmatic trial should be considered a “dress rehearsal” for the intervention to be scaled up at the end of the trial [59]; therefore, ideally, no restrictions should be added to the trial that will not be carried through to usual care once the intervention has been evaluated. Thus, trial planning and conduct should minimise the impact on behaviours of patients, care providers and outcome assessors. In the end, heterogeneity must be considered and accommodated in the planning, conduct and analysis of a trial.

The arguments developed in the present paper represent the opinions of the authors and are not based on original material or systematic reviews. However, all authors are familiar with randomised trials: they all have been involved in many randomised trials and have conducted methodological work in this field. Therefore, these recommendations rely on personal experiences to date, and we acknowledge that they will need to be updated as knowledge of pragmatic approaches to randomised trials evolves. Indeed, pragmatic trials have received much attention over the last years, although the seminal paper was published more than 50 years ago. Finally, although trials have long been viewed as pragmatic or not, even this original paper described the situation as more complex. The overall intention of the trial designers can fairly be described as either pragmatic (to produce information for decision-making) or explanatory (to clarify an understanding of the mechanisms of action of an intervention), but most trialists now agree that there exist several domains relating to the design choices within the trial and that pragmatism should be viewed as a continuum rather than a dichotomous feature within each domain [7, 31, 60]. The appropriate design approach for each domain should aim at matching the overall intention while optimising the balance between wanted and unwanted heterogeneity.

## Data Availability

No data were collected.
